# Rapid review: Three ways local government could improve inequality, public health and wellbeing outcomes in supported housing in England

**DOI:** 10.1016/j.puhip.2025.100692

**Published:** 2025-12-15

**Authors:** K. Kennedy, A. Barnes, A. Formby, N. Pleace, K. Pybus, K. Brain, F. Phillips

**Affiliations:** a The Department of Health Sciences at the University of York, Health Determinants Research Collaboration Bradford Policy Hub, UK; b The Department of Health Sciences at the University of York and the Academic Lead of the University of York, Health Determinants Research Collaboration Bradford Policy Hub, UK; c University of York's School for Business and Society, UK; d Hull York Medical School, UK; e University of York, Health Determinants Research Collaboration Bradford Policy Hub, UK

**Keywords:** Supported housing, Rapid review, Housing inequalities

## Abstract

**Objectives:**

To rapidly review evidence of public health, wellbeing, and/or inequality outcomes of different supported housing schemes, with a focus on identifying relevant lessons from the evidence for local government in England.

**Study design:**

Rapid evidence review.

**Methods:**

Peer reviewed qualitative, quantitative and/or mixed methods studies were identified for review. Databases (EMBASE, ASSIA) were searched in September–October 2024. A two-phase screening and selection process was conducted, with papers sifted and ranked for relevance. Data on outcomes, factors, and implications of supported housing related to public health, wellbeing and/or inequality was extracted from papers ranked of highest relevance.

**Results:**

Six key findings were identified: 1) health outcomes (e.g. symptom management, hospitalisation rates) in supported housing vary by type of support and population; 2) there are varied understandings of ‘successful’ outcomes for people who access supported housing: success depends on who is being supported and in what types of supported housing; 3) quality of life outcomes relate to how supported housing is operated and governed, and how support is provided; 4) the quality of the environment (physical housing, social and community) is critical to rehabilitation, life progression and health and wellbeing outcomes; 5) autonomy is clearly linked to resident experience, life progression and health and wellbeing outcomes; and 6) approaches to support and care are currently not addressing all needs nor promoting ‘successful’ care. Trust and relationships are key aspects to building successful care.

**Conclusions:**

As supported housing has been opaque historically in what it is, definitions, and what it is for, this has consequences for the system – therefore we need to be clearer about what the benefits are, and what realistic goals for supported housing should be. Three ways local government in England can improve supported housing are: 1) local government could usefully approach supported housing as a public health asset and link with relevant parties and leverage partnerships to affect change locally; 2) as supported housing is part of a complicated wider local system of service delivery, complexity-informed evaluation is needed to evaluate appropriate outcomes for populations or individuals accessing supported housing; and 3) because care and support approaches do not currently meet all needs, strategic action is needed in the supported housing sector to address both quality (e.g. undertrained staff) and quantity issues (e.g. insufficient amounts of care provided).

## 1 Introduction

Supported housing in England refers to a specific form of “accommodation which is provided alongside care, support or supervision to help people with specific needs to live as independently as possible in the community” [1]. It aims to provide safe and secure housing with appropriate support for people who may have particular health needs, and/or who have experienced difficult living conditions (e.g. people who have experienced homelessness, substance misuse, domestic abuse, mental health problems and/or who may be disabled) [[2], [3], [4]]. Commissioned supported housing provision typically refers to specialist housing (e.g. hostels, refuges, purpose-built buildings and sheltered housing) where there are staff members present for a portion of the day, or on-site, around the clock, in more intensive models [1,5]. The care and accommodation aspects of supported housing may be commissioned, funded and administered by numerous actors; including generalist and specialist Housing Associations, charitable providers, and local authorities; with some Housing Associations mostly focused on commissioned housing albeit this is hard to sustain [1]. These actors often interact with each other, which complicates the supported housing landscape. Furthermore, provision and practice varies across UK national governments, devolved authorities, central government and across English local authorities.

The status of the sector is further complicated by the fact that commissioned supported housing is not typically considered a formal part of the UK health system, nor part of ‘social care’, despite operating at the intersection of both, and with particular importance in addressing integrated health and social care issues such as homelessness. Uncertainty around the exact role of supported housing is creating threats to its funding, and an increasing need to demonstrate its direct or indirect value, for example, in terms of reducing costs or pressures on the health, mental health and social care [6,7]. Supported housing is looked to as a health and social care intervention on one level yet is restricted from being defined as such. Given this, it is important to understand its potential outcomes – something not well demonstrated in the UK.

Exempt accommodation is a category of supported housing which is exempt from particular Housing Benefit provisions [8]; defined as, “a resettlement place; or accommodation provided by a county council, housing association, registered charity or voluntary organisation where that body or person acting on their behalf provides the claimant with care, support or supervision” [9]. In this scenario, housing benefit is payable at potentially a significantly higher rate above local Housing Benefit caps than for someone needing financial assistance with rent but who is receiving an ordinary housing management service [10]. Landlords may charge a surcharged rent attributed to the ‘intensive’ level of housing management service provided [1,11]. Other care service needs are paid for separately in exempt accommodation, sometimes via grants or via service charges directly to residents [11].

There has been recent political momentum in England and legislative changes to improve the quality of this particular type of supported exempt accommodation to ensure that it meets people's health and wellbeing needs given issues that have been raised about: poor quality accommodation and services, lack of resident protections, exploitation of vulnerable people, and the gross misuse of housing benefit for commercial gain [9,10,[12], [13], [14]]. The 2023 Supported Housing (Regulatory Oversight) Act now requires local government in England to create local supported exempt accommodation licensing schemes, to have strategic oversight of and publish a Supported Housing Strategy for the provision of supported exempt accommodation in its area [9,12,15].

Given uncertainties about the outcomes of and the policy priority accorded to supported housing in England, it is important that local government policymakers have access to an evidence base to inform strategic action on this.

A team of academic researchers working within the [REDACTED] were asked by policymakers within Bradford Council to carry out an independent rapid review to inform local policy development that:

• Identified and summarised evidence of public health, wellbeing and/or inequality outcomes for different types of supported housing/support schemes (excluding related programmes such as Housing First [2] which were already well known to our immediate audience for this rapid review).
• Identified factors that underpin the effectiveness in achieving different outcomes.
• Identified potential implications for local government reviews and strategies on supported housing.

## 2 Methods

A rapid review was conducted between September–October 2024 in line with local policymakers’ timelines. Rapid reviews condense systematic review methods, allowing for fast-tracked consolidation of academic literature for policy audiences. Evaluation shows rapid reviews can provide timely and relevant evidence for policymakers [16]. As there is not a defined method for rapid reviews (because review methods are adapted to meet policymaker needs and timelines), relevant Cochrane [17] and Health Policy and Systems guidance [18] was modified to fit the purpose and scope of this work.

### 2.1 Searches and criteria

Consultation with two experts in the supported housing field and one library information specialist was completed ahead of the review to determine scope, databases and search terms. While grey literature in this space is known to the authors, which informs the introduction and discussion, consultation with the library information specialist informed the search terms for the review. The policy audience that was the direct recipients of the work described their existing knowledge of Housing First, and requested information on this be excluded, thus we devised our search to reflect this. The search terms “supported housing” – “Housing First” were employed across two databases (EMBASE (health/medical) and ASSIA (social science). The use of these broad supported housing terms was discussed with and approved with a library information specialist for being useful, given that these are terms of relevance to the UK context and with some relevance to other country and regional contexts (given our aim was to include relevant insights from literature from other countries). We are aware other countries may have more specific terms to refer to supported housing, however, given the policy-focused time constraints of the review, it was not possible to search using all other variants of the term. While this is a limitation of the review, it enabled us to provide timely insights of relevance to public health in practice.

The following inclusion criteria were used [[2], [3], [4]]: a) English-language publications from 2009 onwards; b) peer-reviewed studies of any type (e.g. systematic reviews, trials, longitudinal design, qualitative studies) that reported outcomes of supported housing (or equivalent) on health and wellbeing, for any social group(s), and/or factors that shape outcomes, including inequality issues and c) relevant to England (i.e. defined as other high income contexts of Europe, North America and Australasia).

### 2.2 Screening and selection of articles

Evidence was reviewed in two rounds by one academic, as there was insufficient time and resource for second-checking, as is common in rapid reviews [19]. Round one excluded articles outside scope based on title and abstract. Round two involved sifting the remaining abstracts of articles for ‘relevance and richness’ in relation to the aims of this study. Each paper was scored in relation to the following three categories (maximum score of three per category): 1) how directly comparable the paper's understanding or programme of supported housing related to England's supported housing structure; 2) how focused the paper was on public health, wellbeing and/or inequality outcomes; and 3) if factors were identified on positive health, wellbeing and/or inequality outcomes. Papers cumulatively scoring seven or above were reviewed in full (see Supplementary File 1). All rationales for inclusion and exclusion, and scores were logged.

### 2.3 Data extraction and synthesis

Initially, search results were downloaded into EndNote, checked for duplicates, then exported to Excel, given the software's flexibility and ease of use for sorting. Data from included papers was extracted into Excel sheets, logging: paper type, supported housing programme (with explanation), population group(s), content focus, and geographic location(s). Relevant data was also extracted on: a) health, wellbeing and/or inequality outcomes (e.g. change in tenant's anxiety symptoms or reduced spending on housing scheme); b) public, personal and societal influences and/or factors to public health, wellbeing and/or inequality (e.g. staff perception and Quality of Life factors); and c) public health, wellbeing and/or inequality implications. Extracted data was synthesised narratively in relation to six key themes, which were developed followed a period of data familiarisation.

### 2.4 Quality assessment

Included evidence was quality assessed using a summary of relevant assessment checklists sourced from CASP (Critical Appraisal Skills Programme) [[20], [21], [22], [23]]. Outcomes of the quality assessment are reported in Supplementary File 1.

## 3 Results

**Fig. 1:**
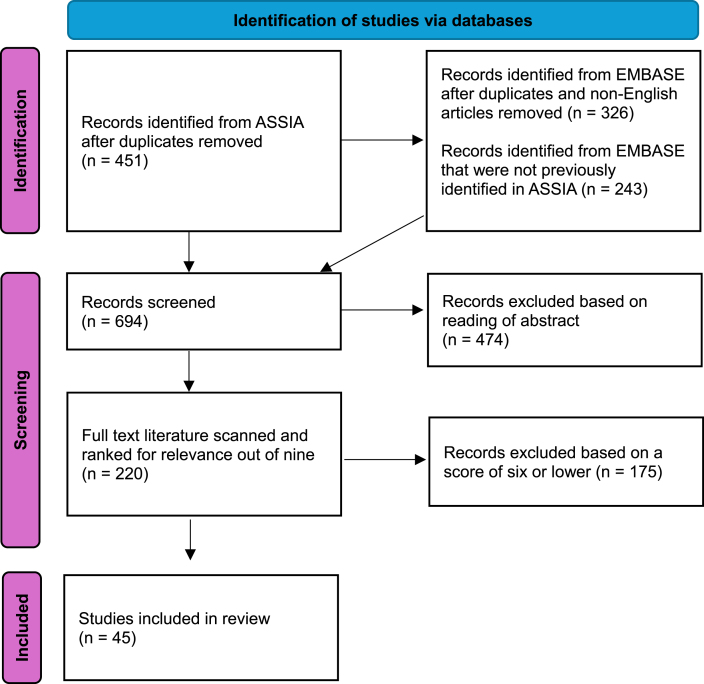
PRISMA Diagram of paper selection process.

In total, 45 articles scored seven or above in the sifting for richness and relevance process and were included in the review: 21 qualitative papers, 10 quantitative, seven mixed-methods and seven systematic reviews (Fig. 1). Within these, 12 population groups were considered in the included literature, with papers most frequently discussing people with severe mental illness (Table 1). The articles included content related to 14 countries, many with multiple appearances across the included papers, most being linked to England and Sweden (Table 2). In part, this country breakdown may reflect the way in which supported housing is conceptualised across different geographies (e.g. supported housing being more common as a distinct sector in North West Europe compared to Eastern Europe) [24].

**Table 1:** Populations specially reviewed in the literature.

Population	Number of papers
**People with severe mental illness**	26
**People with psychiatric disabilities**	8
**People experiencing homeless**	3
**People with intellectual disabilities or are neurodivergent**	3
**Veterans**	2
**Those leaving hospital**	1
**Those abusing substances**	1
**Women facing domestic violence or abuse**	1
**Gypsies and Traveller communities**	1
**People with dementia**	1
**Those engaged in sex work**	1
**Those classed as ex-offenders**	1

12 populations highlighted, with 49 total appearances.

**Table 2:** Countries specially reviewed in the literature.

Country	Number of appearances
**Australia**	5
**Brazil**	1
**Canada**	9
**England**	15
**France**	1
**Germany**	1
**Hong Kong**	1
**Italy**	2
**Netherlands**	4
**Northern Ireland**	2
**Norway**	1
**Sweden**	13
**Switzerland**	2
**USA**	8

14 countries, with 65 total appearances.

### 3.1 Summary of findings

Six key findings were identified in relation to public health, wellbeing and/or inequality outcomes of supported housing.

1 Health outcomes (e.g. symptom management, hospitalisation rates) in supported housing vary by type of support and population

Health outcomes assessed in the literature included general health rates [25], mental health rates, stability/symptom severity [[25], [26], [27]], clinical status [28], and appropriate health service utilisation and self-management [26,29,30]. Mental health improvements were linked to care and support [[25], [26], [27]]. There was minimal data indicating improved physical health outcomes, one paper reported an outcome relating to weight [31]. The lack of reported physical outcomes potentially that physical health changes are not central to supported housing goals nor is it a designated ‘health’ service – i.e. supported housing cannot directly deliver NHS or social care services.

Health outcomes varied depending on which population was studied (e.g. veterans) [25,26,28,32,33] as well as based on the level of supported housing (e.g. high levels of support) [25]. For example, in a supported housing programme in Haringey, London, supporting people with severe mental health problems, general health and mental health rates were highest for those in supported housing forms with high levels of support (e.g. 24 h staffing) compared to medium (regular visits or all-day staff). The worst outcomes were for those with low support (e.g. travelling intermittent staff) [25]. A systematic review reported for those in mental health-centred supported housing, their residence was associated with reduced hospitalisations, increased use of appropriate health services, increased medication visits, and increased use of outpatient clinics [34].

2. There are varied understandings of ‘successful’ outcomes for people who access supported housing: success depends on who is being supported and in what types of supported housing

Duration of residence, previous housing experiences, supported housing conditions and operations, and health and social care integration all shape ‘successful’ outcomes (i.e. thriving within or transitioning out) [26,33,35,36]. Multiple studies identified that ‘move on’ was commonly back into supported housing or another form of it, rather than moving to independent accommodation [31,32]; this can be a designated outcome for some services/models but not always. Durations in supported housing forms also varied, with the literature showing people may not move in the expected time frame, potentially reflecting service ineffectiveness [31]; one study reported that 33 % of residents had moved into the supported accommodation from another supported housing facility, staying approximately 2 years [32]. Durations also can be linked to lack of suitable, affordable, adequate housing options, meaning move on is not possible and residents a ‘stuck’ in the system [37]. Inability to move on due to inadequate housing supply can potentially shift the relationship from being a positive intervention to one that becomes inappropriate or potentially detrimental.

Research varied on the benefits and drawbacks of short versus long term stays. One English study found for each additional year of stay over the expected timeframe, the therapeutic cultural environment within the housing diminished [38]. Other work associated longer durations with having the time to build life skills, engage in training and build confidence, thus supporting more effective outcomes [36].

3. Quality of life outcomes are related to how the supported housing is operated and governed, and how support is provided

Quality of life (QoL) assessments were prevalent in included papers including survey-based assessment toolkits [32] and interviews and observations [39]. While connections were identified in QoL outcomes to different types of supported housing structures when compared (high support, medium support, or floating outreach), evidence was mixed and inconsistent as to which enabled the highest QoL [25,26,32,39,40]. Evidence demonstrated QoL scores can change during the duration of a residency and afterwards. In some literature QoL scores continued to rise throughout this full sequence for those with intellectual disabilities [41] whereas QoL stagnated or reverted for others from homeless groups [26].

4. The quality of the environment (physical housing, social and community) is critical to rehabilitation, life progression and health and wellbeing outcomes

Numerous studies focused on the importance of three aspects of the supported housing environments - physical housing, social environment and community/neighbourhoods – for rehabilitation, life progression, and health and wellbeing outcomes. For physical housing, the structures of supported housing and its maintenance were linked to residents finding meaning in life and satisfaction with living conditions, lower mental health service costs and greater residential stability [30,40,42]. The structure of residents’ environment and ability to create a home environment was highlighted, linked to identity and safety [36,43,44].

The social environment and relationships were highlighted in the literature as important to life progression by building new valuable social networks, combating loneliness and impacting social functioning [33,43,45]. Supported housing can support building social connections outside of family bonds with other residents [33,[46], [47], [48]], but can be taxing [44]. Many residents from various studies struggling with feeling ‘cut off’, having minimal relationships [33,48,49].

For community and neighbourhood, housing stability for residents was linked to the quality of the neighbourhood [26] but often supported housing is located in potentially problematic or unsafe neighbourhoods or in buildings with issues [45,50]. Deterioration in physical quality of the neighbourhood could heighten mental health problems [45]. Community integration widened the potentially narrow world that residents may have in supported housing (e.g. by meeting new people, learning about community amenities, joining activities) and was linked to rehabilitation [27,33,43,46,51].

5. Autonomy is clearly linked to resident experience, life progression and health and wellbeing outcomes

Numerous studies strongly connected autonomy (the ability to control, influence and make independent decisions) to supported housing outcomes, residents' experiences, and sense of identity [25,26,29,39,40,45,46]. Lack of autonomy, choice, and control reduced QoL scores [26,52] and were linked with lower social functioning scores [25]. Control included aspects of how residences operated, such as forced changes in shared spaces and/or where spaces were under staff surveillance [45,53]. Privacy and control over the residents' space and time fed into staff-resident power dynamics akin to ‘mini-institutions’ [45]. However, workers honoured a resident's right to self-determination but found it difficult when residents made short-term choices that may have negative consequences [54].

A 2016 study of the Sicily Group Apartments found, amongst other findings, the cooperative form of supported housing with democratic principles allowed for resident empowerment [30]. The democratic elements of the support housing (e.g. daily morning community meetings where advice and activity planning took place), created room for residents to have autonomy over their lives and space, as well as participation in the community [30]. The approach, besides promoting autonomy, also was found to deliver a more appropriate structure for people with mental health problems in Sicily than larger institutions, with more affordable and appropriate treatment [30].

6. Approaches to support and care are currently not addressing all needs nor promoting ‘successful’ care. Trust and relationships are key aspects to building successful care

Included studies repeatedly highlighted unmet care needs (around form and function, role clarity, and system integration in the supported housing system) and were at all levels of support [25] for multiple populations. This included for adults with intellectual disabilities, homelessness groups, and those with several mental illnesses [25,32,55].

Studies identified the pivotal importance of relationships between support workers and residents; including the need for positive interactions, trust, non-judgemental approaches, and for residents to have autonomy and control to their support and care [42,43,56]. Where trust was established, participants reported positive views about care staff and examples of effective care [37,43,51,56]. How care was integrated into residents’ lives influenced outcomes, including if care was provided internally or externally [26,33,43] and impacted efficacy and experience of support and care [26,52,55,57,58]. Limited staff/worker capacity and skill limitations actively worked against care and life progression [53,58]. Increased staff time and resources for skill development and training was identified as a need in the data [51,52].

## 4 Discussion

This rapid review aimed to identify ways local government in England could improve outcomes for people living in supported housing and inform local government approaches to their changing responsibilities for exempt supported housing under new legislation. As noted in the introduction, the supported housing sector is not typically considered a formal part of the UK health system, nor part of ‘social care’, with this uncertainty around its role creating threats to its funding and a need to demonstrate its value. This review builds towards better understanding and clarity as to the benefits, and what realistic goals for supported housing should be. Included evidence highlighted a range of health, wellbeing and inequalities issues and outcomes in supported housing. Issues related to the quality of housing and support, environment, and autonomy [[25], [26], [27],^31^,^32^,^39^,^40^,^45^,^46^] as well as implications for mental health, housing ‘move on’ and hospitalisation rates [25,27,[30], [31], [32], [33], [34]].

Although the review was carried out rapidly, with a consequent risk that some relevant evidence was not included, we identified three key ways local government can improve outcomes in supported housing in England, as discussed below, which all resonate closely with wider literature on this topic. While the review does not represent a comprehensive review of the literature (i.e. it did not include all papers on the topic), the three areas of potential action for local government came from articles that were judged to be of high richness and relevance and met our quality appraisal criteria for acceptability and rigour.

1. Local government could usefully approach supported housing as a public health asset and link with relevant parties and leverage partnerships to affect change locally

In this review, we identified six key findings related to public health, wellbeing and/or inequality outcomes in supported housing to which we derive lessons local government may find relevant to their engagement with the sector. As noted above, the findings highlight a range of wellbeing, inequality, and health issues within supported housing, including around the environment, QoL and support. To address these, we suggest that local government could usefully approach supported housing as a public health asset and leverage multi-dimensional partnerships across local government, and health and social care to affect change locally [1].

Given the issues highlighted with how care was integrated into people's lives, as well as the importance of autonomy for residents, partners could usefully include NHS providers, including in primary care, supported housing providers, social workers, adult social care providers, charities engaged in the sector and, importantly, those with lived or living experience of supported housing [1,59]. Involvement of those with lived experiences is crucial to ensure that local actions support autonomy and personal dignity, as seen in the Blackburn supported housing pilot, which examined lived experience via a partnership with Shelter [60]. Moreover, some people in supported housing may not be best served by what the sector can offer but have arrived there based on inefficiencies or gaps in services elsewhere in local authorities, and/or a lack of housing supply [5,11,59]. Local government may look to create mechanisms with partners – including residents – to ensure those in supported housing are best placed there rather than engaging with other services [59].

Within this kind of public health partnership approach, partners could consider the value of reviewing their local supported housing landscape in relation to how it addresses poor health and wellbeing outcomes and inequalities, to inform actions to prevent their (re)production. This suggested approach to partnering complements the outcomes of the evaluation of pilot initiatives to improve supported housing quality and value for money [60] and the subsequent local authority guidance from the then Department for Levelling Up, Housing and Communities (e.g. Health and Social Care Partnerships in Scotland) [1,59].

2. As supported housing is part of a complicated wider local system of service delivery, complexity-informed evaluation is needed to evaluate appropriate outcomes for populations or individuals accessing supported housing

Given the complexity of the supported housing landscape and how its provision intersects with wider health and social care system in England [25,61], there needs to be appreciation and considerations of the complexities. Within any partnership approach, complexity-informed approaches to evaluation and assessment are needed to enable ‘fit for purpose’ strategic reviews of local provision of exempt supported housing and service planning [60], in part relating to the evolving role of local government. Complexity-informed approaches are important because they take account of a range of evidence, and can support discussion and learning about the trade-offs between different definitions of success in supported housing for different population groups and individuals [40,[62], [63], [64]]. Measures of success need to be tailored to the specific needs of the service user group. Service planning to achieve these and evaluative approaches need to take account of this, as well as non-linear journeys, recognising that moves in and out of supported housing and from one type of supported housing to another are common: data and evidence collection needs to reflect this reality.

3. Because care and support approaches do not currently meet all needs, strategic action is needed in the supported housing sector to address both quality (e.g. undertrained staff) and quantity issues (e.g. insufficient amounts of care provided)

Our findings clearly highlighted that existing care and support approaches in supported housing do not currently meet all needs. In England, this particularly may be the case as there is a not a legal or clear definition as to what compromises care, support and supervision [1]. While clarity here will require national action, strategic local action is also important to more fully understand where needs are not being met, including through approaches that consider the “whole environment” (i.e. how the physical quality of accommodation, the social communities of service users and the wider neighbourhoods/communities affect health and well-being outcomes), the quality of the relationships between service users and support professionals, and value resident autonomy and influence in decision-making [5]. Supported housing alone cannot be expected, to provide care and support residents to thrive: a multi-sectoral public health approach, including to intersecting issues, such as homelessness, is needed [65]. To this end, local government and its partners could usefully ensure that all relevant strategic boards that focus on intersecting issues with the sector (e.g. Health and Wellbeing Board, Community Safety Partnerships) are aware of the challenges and complexity of supported housing issues and are involved in strategic reviews and action to better meet local needs. Local guidance and guidelines for what constitutes ‘good support’, could usefully be co-produced with a range of actors, including residents [66], and mobilised for action by these strategic boards. Strategic, coordinated action of this kind is important because, without intervention, poor outcomes will have a knock-on effect on demand for services across local health and social care systems [26,29,30].

## Funding

This work was completed as part of the National Institute for Health and Care Research Health Determinants Research Collaboration Bradford. NIHR Health Determinants Research Collaboration Bradford is funded by the PHR Programme (NIHR151305). The views expressed are those of the author(s) and not necessarily those of the NIHR or the Department of Health and Social Care. Kennedy, Barnes, and Formby are funded by the University of York Research Development Funds. The York Policy Engine is supported by the UKRI Research England Development Fund.

## Ethical statement

As this rapid review did not involve human participants, no ethical approval was required.

## Declaration of competing interest

The authors declare the following financial interests/personal relationships which may be considered as potential competing interests.

K. Kennedy is a research associate to the University of York/Health Determinants Research Collaboration Bradford Policy Hub and was involved in helping to scope up the protocol for this evidence review, carried out the review in full and prepared the manuscript.

A. Barnes is an academic lead of the University of York/Health Determinants Research Collaboration Bradford Policy Hub and was involved in scoping up the local policy and practice need for this evidence review and provided feedback on the findings/manuscript.

A. Formby is a policy follow to the University of York/Health Determinants Research Collaboration Bradford Policy Hub and was involved in scoping up the local policy and practice need for this evidence review and provided feedback on the findings/manuscript.

N. Pleace is a professor at the University of York's School for Business and Society and consulted on the protocol of the rapid review design and provided feedback on the findings/manuscript.

K. Pybus is a lecturer at Hull York Medical School and consulted on the protocol on the rapid review design and provided feedback on the findings/manuscript.

K. Brain is a Bradford Council lead for the University of York/Health Determinants Research Collaboration Bradford Policy Hub was involved scoping up the local policy and practice need for this evidence review and provided feedback on the findings/manuscript.

F. Phillips is a Bradford Council lead for the University of York/Health Determinants Research Collaboration Bradford Policy Hub was involved scoping up the local policy and practice need for this evidence review and provided feedback on the findings/manuscript.
