# Supplementary material for: Rapid review: Three ways local government could improve inequality, public health and wellbeing outcomes in supported housing in England
Source: Public Health Pract (Oxf). 2025 Dec 15;11:100692. doi: 10.1016/j.puhip.2025.100692 (PMC12818142; doi:10.1016/j.puhip.2025.100692)
Supplement: Multimedia component 1 [file mmc1.docx]

# Supplementary File

Table 1: Richness Score Reporting Summary

| **Paper (out of 45)** | **Name** | **Author** | **Year** | **Richness Criteria 1: How directly comparable to Supported Housing in England? 3 = like for like/exact; 2 = notable overlap; 1 = slight or no overlap** | **Richness Criteria 2: How focused on public health, wellbeing, and/or inequality outcomes is the paper? 3 = outcome driven; 2 = has some outcomes; 1 = little to no outcome focus** | **Richness Criteria 3: Are factors identified on positive health, wellbeing and/or inequality outcomes? 3 = factor(s) stated; 2 = factor(s) clearly alluded to or derivable; 1 = little to no mention** | **Overall Richness Criteria score (out of 9):** |
| --- | --- | --- | --- | --- | --- | --- | --- |
| 1 | Quality of life, autonomy, satisfaction, and costs associated with mental health supported accommodation services in England: a national survey | Killaspy et al. | 2016 | 3 | 3 | 2 | 8 |
| 2 | Quality of life outcomes for people with serious mental illness living in supported accommodation: systematic review and meta-analysis | Harrison et al. | 2020 | 3 | 3 | 2 | 8 |
| 3 | Quality of life and unmet need in people with psychosis in the London Borough of Haringey, UK | Lambri et al. | 2012 | 3 | 3 | 2 | 8 |
| 4 | Mental health supported accommodation services: A systematic review of mental health and psychosocial outcomes | McPherson et al. | 2018 | 2 | 3 | 3 | 8 |
| 5 | Significant others' perspectives on experiences of meal-oriented support and diet counselling for adults with intellectual disabilities who live in supported housing | Adolfsson et al. | 2022 | 3 | 2 | 3 | 8 |
| 6 | Not Going It Alone: Social Integration and Tenancy Sustainability for Formerly Homeless Substance Users | Bowpitt and Harding | 2009 | 3 | 2 | 3 | 8 |
| 7 | Striving for meaning-Life in supported housing for people with psychiatric disabilities | Brolin et al. | 2016 | 3 | 2 | 3 | 8 |
| 8 | Group-apartments for recovery of people with psychosis in Italy: Democratic therapeutic communities in post-modern social communities | Bruschetta and Barone | 2016 | 3 | 3 | 2 | 8 |
| 9 | The Resettlement of Homeless Young People: Their Experiences and Housing Outcomes | Crane et al. | 2014 | 3 | 3 | 2 | 8 |
| 10 | Outcomes of the Active in My Home (AiMH) intervention for people with psychiatric disabilities in supported housing: A longitudinal pilot and feasibility study | Eklund et al. | 2020 | 3 | 3 | 2 | 8 |
| 11 | Studies regarding supported housing and the built environment for people with mental health problems: A mixed-methods literature review | Friesinger et al. | 2019 | 3 | 3 | 2 | 8 |
| 12 | Towards an integrated approach to homeless hospital discharge: Managing Community Care | Albanese et al. | 2016 | 2 | 3 | 2 | 7 |
| 13 | Client Experiences With Shelter and Community Care Services in the Netherlands: Quality of Services for Homeless People, Homeless Youth, and Abused Women | Asmoredjo et al. | 2017 | 3 | 2 | 2 | 7 |
| 14 | Residents' perceptions of the most positive and negative aspects of the housing situation for people with psychiatric disabilities | Brolin et al. | 2018 | 3 | 2 | 2 | 7 |
| 15 | Do Home-Based Psychiatric Services for Patients in Medico-Social Institutions Reduce Hospitalizations? Pre-Post Evaluation of a French Psychiatric Mobile Team | Cervello et al. | 2019 | 3 | 1 | 3 | 7 |
| 16 | Transitional supported housing for mental health consumers enabling personal recovery: Allowing me to be me | Dorozenko et al. | 2018 | 3 | 2 | 2 | 7 |
| 17 | The effects of supported housing for individuals with mental disorders | Lopes et al. | 2022 | 2 | 3 | 2 | 7 |
| 18 | Navigating in a Misty Landscape – Perceptions of Supporting a Relative Residing in Supported Housing for People with a Psychiatric Disability | Gunnarsson et al. | 2020 | 3 | 1 | 3 | 7 |
| 19 | The Accommodation Experiences of Older Gypsies and Travellers: Personalisation of Support and Coalition Policy | Hodges and Cemlyn | 2013 | 3 | 2 | 2 | 7 |
| 20 | Predictors of moving on from mental health supported accommodation in England: national cohort study | Killaspy et al. | 2020 | 3 | 3 | 1 | 7 |
| 21 | Effects of Social Networks on Physical Health Among People with Serious Mental Illness | Lee et al. | 2014 | 2 | 2 | 3 | 7 |
| 22 | Home Bittersweet Home: the Significance of Home for Occupational Transformations | Lindström et al. | 2011 | 2 | 2 | 3 | 7 |
| 23 | Stories of Rediscovering Agency: Home-Based Occupational Therapy for People With Severe Psychiatric Disability | Lindstrom et al. | 2013 | 2 | 2 | 3 | 7 |
| 24 | Support in Daily Living for Young Adults with Neurodevelopmental Conditions in Sweden: A Qualitative Description of Current Practice | Löthberg et al. | 2024 | 3 | 1 | 3 | 7 |
| 25 | The Influence of Physical Environmental Qualities on the Social Climate of Supported Housing Facilities for People with Severe Mental Illness | Marcheschi et al. | 2013 | 3 | 1 | 3 | 7 |
| 26 | From traditional counselling to health‐promoting conversations? Registered nurses' experiences of providing health counselling to people living with severe mental ill‐health in supported housing | Molin et al. | 2023 | 3 | 1 | 3 | 7 |
| 27 | 'I didn't like just sittin' around all day': Facilitating Social and Community Participation Among People with Mental Illness and High Levels of Psychiatric Disability | Muir et al. | 2010 | 3 | 2 | 2 | 7 |
| 28 | Direct Placement Versus Multistage Models of Supported Housing in a Population of Veterans Who Are Homeless | O'Connell et al. | 2009 | 2 | 3 | 2 | 7 |
| 29 | Supported housing for adults with psychiatric disabilities: How tenants confront the problem of loneliness | Piat et al. | 2018 | 3 | 1 | 3 | 7 |
| 30 | Supported housing for persons with serious mental illness and personal recovery: What do families think? | Piat and Seida | 2018 | 3 | 2 | 2 | 7 |
| 31 | Choice and personal recovery for people with serious mental illness living in supported housing | Piat et al. | 2020 | 3 | 1 | 3 | 7 |
| 32 | Understanding everyday life and mental health recovery through CHIME | Piat et al. | 2017 | 3 | 1 | 3 | 7 |
| 33 | Evaluating quality of life in adults with profound learning difficulties resettled from hospital to supported living in the community | Sines et al. | 2012 | 2 | 3 | 2 | 7 |
| 34 | Living with dementia in supported housing: A systematic review and thematic synthesis of qualitative research | Smith et al. | 2022 | 3 | 2 | 2 | 7 |
| 35 | A home but how to connect with others? A qualitative meta‐synthesis of experiences of people with mental illness living in supported housing | Watson et al. | 2019 | 3 | 2 | 2 | 7 |
| 36 | The Impacts of Intersecting Stigmas on Health and Housing Experiences of Queer Women Sex Workers in Vancouver, Canada | Lyons et al. | 2021 | 3 | 2 | 2 | 7 |
| 37 | A qualitative study of how people with severe mental illness experience living in sheltered housing with a private fully equipped apartment | Roos et al. | 2016 | 2 | 2 | 3 | 7 |
| 38 | Psychosocial functioning of individuals with schizophrenia in community housing facilities and the psychiatric hospital in Zurich | Jaeger et al. | 2015 | 3 | 3 | 1 | 7 |
| 39 | Predictors of quality of care in mental health supported accommodation services in England: A multiple regression modelling study | Dalton-Locke et al. | 2018 | 3 | 2 | 2 | 7 |
| 40 | A place to live: Housing needs for people with psychotic disorders identified in the second Australian national survey of psychosis | Harvey et al. | 2012 | 3 | 2 | 2 | 7 |
| 41 | A place to call home: perspectives on offender community reintegration | Tarpey and Friend | 2016 | 3 | 1 | 3 | 7 |
| 42 | Patient and program predictors of 12-month outcomes for homeless veterans following discharge from time-limited residential treatment | McGuire et al. | 2011 | 2 | 3 | 2 | 7 |
| 43 | Lifestyle interventions for people with a severe mental illness living in supported housing: A systematic review and meta-analysis | Koomen et al. | 2022 | 2 | 3 | 2 | 7 |
| 44 | Intentions and experiences of effective practice in mental health specific supported accommodation services: a qualitative interview study | Sandhu et al. | 2017 | 3 | 1 | 3 | 7 |
| 45 | Housing services for people with mental disorders in England: Patient characteristics, care provision and costs | Priebe et al. | 2009 | 2 | 2 | 3 | 7 |

Table 2: Quality Assessment Reporting Summary – Adapted from CASP checklists

| **Paper (out of 25)** | **Name** | **Author** | **Year** | **Are the results of the study valid? (e.g. was there a clear research statement of the aims, was the methodology appropriate, was the recruitment strategy appropriate)**  **Y= Yes; N = No; U = Unsure/Can’t Tell** | **What are the results? (e.g. is there a clear statement of findings, are ethical issues taken into consideration, was the data analysed to a sufficient level of rigour?)**  **Y= Yes; N = No; U = Unsure/Can’t Tell** | **Will the results help locally? (e.g. is the research valuable?)**  **Y= Yes; N = No; U = Unsure/Can’t Tell** | **Appraisal notes, consideration comment or summary statement** |
| --- | --- | --- | --- | --- | --- | --- | --- |
| 1 | Quality of life, autonomy, satisfaction, and costs associated with mental health supported accommodation services in England: a national survey | Killaspy et al. | 2016 | Y | Y | Y | Article detailed in all areas of consideration. |
| 2 | Quality of life outcomes for people with serious mental illness living in supported accommodation: systematic review and meta-analysis | Harrison et al. | 2020 | Y | Y | Y | High level of detail regarding methods and limitations. |
| 3 | Quality of life and unmet need in people with psychosis in the London Borough of Haringey, UK | Lambri et al. | 2012 | Y | Y | Y | Includes both resident and care-workers’ assessments of need. |
| 4 | Mental health supported accommodation services: A systematic review of mental health and psychosocial outcomes | McPherson et al. | 2018 | Y | Y | Y | Used the Quality Assessment Tool for Quantitative Studies for their article quality assessment. |
| 5 | Significant others' perspectives on experiences of meal-oriented support and diet counselling for adults with intellectual disabilities who live in supported housing | Adolfsson et al. | 2022 | Y | Y | Y | While it is a quality article and has key takeaways that apply to the English context, the entirety of the work may not be applicable – a limitation acknowledged in the study. |
| 6 | Not Going It Alone: Social Integration and Tenancy Sustainability for Formerly Homeless Substance Users | Bowpitt and Harding | 2009 | Y | Y | Y | While the findings are valid overall, there are some weaknesses in the recruitment and methodological reporting. |
| 7 | Striving for meaning-Life in supported housing for people with psychiatric disabilities | Brolin et al. | 2016 | Y | Y | Y | Clear description of their use of classic grounded theory. |
| 8 | Group-apartments for recovery of people with psychosis in Italy: Democratic therapeutic communities in post-modern social communities | Bruschetta and Barone | 2016 | U | Y | Y | Would need to further investigate the studies discussed in section 6 to fully assess validity. |
| 9 | The Resettlement of Homeless Young People: Their Experiences and Housing Outcomes | Crane et al. | 2014 | Y | Y | Y | Draws from the larger FOR-HOME study |
| 10 | Outcomes of the Active in My Home (AiMH) intervention for people with psychiatric disabilities in supported housing: A longitudinal pilot and feasibility study | Eklund et al. | 2020 | Y | Y | Y | Clearly reported the limitations and value of its findings. |
| 11 | Studies regarding supported housing and the built environment for people with mental health problems: A mixed-methods literature review | Friesinger et al. | 2019 | Y | Y | Y | Clear outlining of approach to the literature search. |
| 12 | Towards an integrated approach to homeless hospital discharge: Managing Community Care | Albanese et al. | 2016 | Y | Y | Y | Did acknowledge limitation that the data was self-reported and quality/completeness varied. |
| 13 | Client Experiences With Shelter and Community Care Services in the Netherlands: Quality of Services for Homeless People, Homeless Youth, and Abused Women | Asmoredjo et al. | 2017 | Y | Y | Y | Detailed methods and data collection section. |
| 14 | Residents' perceptions of the most positive and negative aspects of the housing situation for people with psychiatric disabilities | Brolin et al. | 2018 | Y | Y | Y | Discusses the role of project assistants and how that influenced recruitment. |
| 15 | Do Home-Based Psychiatric Services for Patients in Medico-Social Institutions Reduce Hospitalizations? Pre-Post Evaluation of a French Psychiatric Mobile Team | Cervello et al. | 2019 | Y | Y | Y | Data available to request from author. |
| 16 | Transitional supported housing for mental health consumers enabling personal recovery: Allowing me to be me | Dorozenko et al. | 2018 | Y | Y | Y | Includes reflexivity and checking data and interpretations with participants. |
| 17 | The effects of supported housing for individuals with mental disorders | Lopes et al. | 2022 | Y | Y | Y | Secondary analysis of pseudonymised administrative data. |
| 18 | Navigating in a Misty Landscape – Perceptions of Supporting a Relative Residing in Supported Housing for People with a Psychiatric Disability | Gunnarsson et al. | 2020 | Y | Y | Y | Specific section on the methodological considerations, e.g. trustworthiness, credibility, and transferability. |
| 19 | The Accommodation Experiences of Older Gypsies and Travellers: Personalisation of Support and Coalition Policy | Hodges and Cemlyn | 2013 | Y | Y | Y | Only two of the eight user interviews went ahead, however other data sources, including use of secondary data, utilised. |
| 20 | Predictors of moving on from mental health supported accommodation in England: national cohort study | Killaspy et al. | 2020 | Y | Y | Y | Detailed list of limitations. |
| 21 | Effects of Social Networks on Physical Health Among People with Serious Mental Illness | Lee et al. | 2014 | Y | Y | Y | The focus on social networks has relevance and support, form of housing should be considered though in how it’s used compared to England. |
| 22 | Home Bittersweet Home: the Significance of Home for Occupational Transformations | Lindström et al. | 2011 | Y | Y | Y | Methodological considerations about interviewer’s familiarity with residents. |
| 23 | Stories of Rediscovering Agency: Home-Based Occupational Therapy for People With Severe Psychiatric Disability | Lindstrom et al. | 2013 | Y | Y | Y | Article from part of the larger 2007 study. |
| 24 | Support in Daily Living for Young Adults with Neurodevelopmental Conditions in Sweden: A Qualitative Description of Current Practice | Löthberg et al. | 2024 | Y | Y | Y | Purposive sampling framework used – detailed. |
| 25 | The Influence of Physical Environmental Qualities on the Social Climate of Supported Housing Facilities for People with Severe Mental Illness | Marcheschi et al. | 2013 | Y | Y | Y | Acceptable but must consider the limitations as to not overrepresent its applicability and validity. |
| 26 | From traditional counselling to health‐promoting conversations? Registered nurses' experiences of providing health counselling to people living with severe mental ill‐health in supported housing | Molin et al. | 2023 | Y | Y | Y | Includes a paragraph outlining relevance for clinical practice. |
| 27 | 'I didn't like just sittin' around all day': Facilitating Social and Community Participation Among People with Mental Illness and High Levels of Psychiatric Disability | Muir et al. | 2010 | Y | Y | Y | Commissioned evaluation of HASI. |
| 28 | Direct Placement Versus Multistage Models of Supported Housing in a Population of Veterans Who Are Homeless | O'Connell et al. | 2009 | Y | Y | U | Use with caution, as the results may not apply seamlessly locally. |
| 29 | Supported housing for adults with psychiatric disabilities: How tenants confront the problem of loneliness | Piat et al. | 2018 | Y | Y | Y | Clear top level statements of ‘what this paper adds’ |
| 30 | Supported housing for persons with serious mental illness and personal recovery: What do families think? | Piat and Seida | 2018 | Y | Y | Y | Geographic limitations in the recruitment of family members to participate. |
| 31 | Choice and personal recovery for people with serious mental illness living in supported housing | Piat et al. | 2020 | Y | Y | Y | Gives scale details of the supported housing projects recruited from. |
| 32 | Understanding everyday life and mental health recovery through CHIME | Piat et al. | 2017 | Y | Y | Y | Application of the CHIME framework, utilising go-along technique. |
| 33 | Evaluating quality of life in adults with profound learning difficulties resettled from hospital to supported living in the community | Sines et al. | 2012 | Y | Y | Y | Quality of Life Questionnaire V3 required a ‘train the trainer’ approach for audit. |
| 34 | Living with dementia in supported housing: A systematic review and thematic synthesis of qualitative research | Smith et al. | 2022 | Y | Y | Y | Has quality appraisal using CASP. |
| 35 | A home but how to connect with others? A qualitative meta‐synthesis of experiences of people with mental illness living in supported housing | Watson et al. | 2019 | Y | Y | Y | Includes quality appraisal. |
| 36 | The Impacts of Intersecting Stigmas on Health and Housing Experiences of Queer Women Sex Workers in Vancouver, Canada | Lyons et al. | 2021 | Y | Y | U | Cohort methods detailed in other work referenced. Also need to be cognisant of varying experiences of housing of cohort. |
| 37 | A qualitative study of how people with severe mental illness experience living in sheltered housing with a private fully equipped apartment | Roos et al. | 2016 | Y | Y | Y | Includes a strengths and limitations section which should be noted. |
| 38 | Psychosocial functioning of individuals with schizophrenia in community housing facilities and the psychiatric hospital in Zurich | Jaeger et al. | 2015 | Y | Y | Y | Includes clinical and demographic characteristic of residents and acute inpatients. |
| 39 | Predictors of quality of care in mental health supported accommodation services in England: A multiple regression modelling study | Dalton-Locke et al. | 2018 | Y | Y | Y | In results addressing the missing data issue. |
| 40 | A place to live: Housing needs for people with psychotic disorders identified in the second Australian national survey of psychosis | Harvey et al. | 2012 | Y | Y | Y | Key limitation was a likely underestimate of those experiencing homelessness and experiencing psychosis. |
| 41 | A place to call home: perspectives on offender community reintegration | Tarpey and Friend | 2016 | Y | Y | Y | Small sample size n=5. |
| 42 | Patient and program predictors of 12-month outcomes for homeless veterans following discharge from time-limited residential treatment | McGuire et al. | 2011 | Y | Y | Y | Key concern over veterans not being randomly assigned to programme type. |
| 43 | Lifestyle interventions for people with a severe mental illness living in supported housing: A systematic review and meta-analysis | Koomen et al. | 2022 | Y | Y | Y | Used PRISMA and conducted quality assessment. |
| 44 | Intentions and experiences of effective practice in mental health specific supported accommodation services: a qualitative interview study | Sandhu et al. | 2017 | Y | Y | Y | Details the cross-coding process in thematic analysis. |
| 45 | Housing services for people with mental disorders in England: Patient characteristics, care provision and costs | Priebe et al. | 2009 | Y | Y | Y | Decided against speaking with residents due to bias concerns. |
